# Effects of cardiopulmonary bypass on the disposition of cefazolin in patients undergoing cardiothoracic surgery

**DOI:** 10.1002/prp2.440

**Published:** 2018-11-05

**Authors:** Mizuho Asada, Masashi Nagata, Tomohiro Mizuno, Tokujiro Uchida, Naoki Kurashima, Hiromitsu Takahashi, Koshi Makita, Hirokuni Arai, Hirotoshi Echizen, Masato Yasuhara

**Affiliations:** ^1^ Department of Pharmacy Medical Hospital Tokyo Medical and Dental University (TMDU) Tokyo Japan; ^2^ Department of Pharmacokinetics and Pharmacodynamics Graduate School of Medical and Dental Sciences Tokyo Medical and Dental University (TMDU) Tokyo Japan; ^3^ Department of Cardiovascular Surgery Graduate School of Medical and Dental Science Tokyo Medical and Dental University (TMDU) Tokyo Japan; ^4^ Department of Anesthesiology Graduate School of Medical and Dental Sciences Tokyo Medical and Dental University (TMDU) Tokyo Japan; ^5^ Medical Engineering Center Medical Hospital of Tokyo Medical and Dental University (TMDU) Tokyo Japan; ^6^ Department of Pharmacotherapy Meiji Pharmaceutical University Tokyo Japan

**Keywords:** cardiopulmonary bypass, cefazolin, plasma protein binding, surgical site infection, unbound fraction

## Abstract

The aim of the study was to evaluate the disposition of plasma unbound cefazolin in patients undergoing cardiothoracic surgery with cardiopulmonary bypass (CPB). Adult patients undergoing cardiothoracic surgery with CPB were enrolled in the study. Cefazolin sodium was given intravenously before skin incision (1 g) and at the beginning of CPB (2 g). Thereafter, an additional dose (1 g) was given every 4 hours. Seven to ten blood samples were collected before and during surgery. Plasma total and unbound (ultrafiltrated) cefazolin concentrations were analyzed using an HPLC‐UV method. Plasma protein binding was analyzed with the Langmuir model. Twenty‐seven patients (aged 70 ± 12 years, body weight 62 ± 12 kg, mean ± SD) with GFR >30 mL min^−1^ completed the study. There was a significant (*P* < 0.001) increase in median plasma unbound fraction of cefazolin from 21% before skin incision to 45% during CPB (*P* < 0.001), which was accompanied by a significant (*P* < 0.001) reduction in median plasma albumin concentration from 36 to 27 g L^−1^. Plasma concentrations of unbound cefazolin exceeded the assumed target thresholds of 2 μg mL^−1^ in all samples and of 8 μg mL^−1^ in all but one of 199 samples. The increased plasma unbound fraction of cefazolin would be attributable to dilutional reduction of serum albumin at the beginning of CPB and to saturable plasma protein binding of cefazolin. These data reveal CPB may alter the plasma protein binding and possibly distribution of cefazolin. Further studies are warranted to reappraise the protocol of antimicrobial prophylaxis with cefazolin in patients undergoing surgery with CPB.

**Abbreviations**
CPB: cardiopulmonary bypass
GFR: glomerular filtration rate
HPLC: high performance liquid chromatography
ISF: interstitial fluid
MSSA: methicillin‐sensitive Staphylococcus aureus

## 1 INTRODUCTION

Surgical site infection (SSI) is one of the major postoperative complications and is associated with increased morbidity and mortality.^1^, ^2^ Particularly, SSIs (such as mediastinitis) that develop in patients undergoing cardiothoracic surgery are associated with a high mortality. Previous meta‐analyses revealed that antimicrobial prophylaxis used in cardiothoracic surgery is associated with better surgical outcomes.^3^, ^4^ While cardiothoracic surgery is often performed with cardiopulmonary bypass (CPB), there is a paucity of knowledge about changes that may occur in the pharmacokinetics of drugs during CPB. As a result, consensus has not been attained about the choice and dosing protocols of antibiotics for these patients.^5^ The guidelines of the American Society of Health‐System Pharmacists recommend that traditional antimicrobial prophylaxis protocols for general surgery should not be changed for patients undergoing cardiothoracic surgery with CPB, unless further clinical outcome data obtained from well‐designed studies are available.^1^ Nevertheless, recent studies argued that conventional regimens of cefazolin prophylaxis for these patients may not be reliable in maintaining a target plasma threshold (for example, total drug concentration of 40 μg mL^−1^, assuming normal protein binding of 80%‐86% 6, 7 during CPB) at intraoperative trough and/or at wound closure in patients with normal renal function undergoing cardiac surgery with CPB.^7^

CPB may alter pharmacokinetics of antimicrobial agents by multiple mechanisms. For instance, approximately 1.3 L of crystalloid solution is administered to prime the bypass circuit, and a balanced crystalloid solution is administered prior to and during CPB to expand the circulating volume for maintaining cardiovascular stability, due to blood loss during operation. As a result, substantial dilutional reduction of serum albumin levels (<30 g L^−1^) occurs, and it may increase the volume of distribution and alter plasma protein binding of drugs that have a small volume of distribution and extensive plasma protein binding. In addition, heparin is routinely administered intravenously at 300 to 400 U kg^−1^ into the CPB circuit for anticoagulation. A heparin‐induced increase in plasma free fatty acids may competitively displace drugs from their albumin‐binding sites.^8^ Furthermore, CPB may affect systemic elimination of drugs by altering hepatic and/or renal blood flow.^9^, ^10^

Current guidelines recommend intravenous cefazolin sodium as the drug of choice for antimicrobial prophylaxis in patients undergoing surgery, due to its excellent activity against common pathogens causing SSI.^1^, ^11^ Since cefazolin binds extensively to albumin (80%‐86%) 6, 7 and has a small volume of distribution (10 L per body),^6^ its disposition may be particularly susceptible to CPB‐induced physiological changes. Indeed, previous studies reported a 28%‐50% decrease in plasma total cefazolin concentration during CPB.^12^, ^13^ Collectively, there is a paucity of knowledge about the impact of CPB on the disposition of cefazolin. Here, we report the disposition of total and unbound cefazolin throughout cardiothoracic surgery performed with CPB, with reference to the target plasma unbound drug concentration.

## 2 MATERIALS AND METHODS

### 2.1 Patients and CPB procedures

Adult patients who underwent cardiothoracic surgery with CPB at the Medical Hospital, Tokyo Medical and Dental University between May 2015 and July 2017 were enrolled in the study. The protocol of the present study was approved by the Ethics Committee of the Faculty of Medicine, Medical Research Tokyo Medical and Dental University, Tokyo, Japan before the study was begun (authorization number: M2000‐1895). Prior to participation in the study, each patient provided written informed consent. Exclusion criteria were a history of hypersensitivity to cephalosporins or penicillins, comorbidities of severe renal dysfunction [estimated glomerular filtration rate (GFR) < 30 mL min^−1^] and chronic liver diseases, active infection at the time of surgery, and antimicrobial treatment during one week prior to operation. GFR was estimated (eGFR) according to the equation established for Japanese 14:

$$\begin{array}{cc} & \mathrm{eGFR}=194\times {\left[\mathrm{serum}\;\;\mathrm{creatinine}\right]}^{-1.094}\\ & \times {\left[\mathrm{age}\right]}^{-0.287}\times \frac{\mathrm{body}\;\;\mathrm{surface}\;\;\;\mathrm{area}}{1.73}\;\;\;\left(\mathrm{mL}\;\;\;\;\min^{-1}\right)\;\;\mathrm{for}\;\;\mathrm{men} \end{array}$$

$$\begin{array}{cc} \mathrm{eGFR} & =194\times {\left[\mathrm{serum}\;\;\mathrm{creatinine}\right]}^{-1.094}\\ & \times {\left[\mathrm{age}\right]}^{-0.287}\times \frac{\mathrm{body}\;\;\mathrm{surface}\;\;\;\mathrm{area}}{1.73}\times 0.739\;\;\;\left(\mathrm{mL}\;\;\;\;\min^{-1}\right)\;\;\mathrm{for}\;\;\mathrm{women} \end{array}$$

CPB was performed using the Advanced Perfusion System 1 (Terumo, Tokyo, Japan). The CPB circuit was primed with 1100‐1950 mL of fluid containing 2 mL kg^−1^ mannitol, 500 mL of hydroxyethyl starch products, and approximately 500 mL of lactated Ringer solution. During CPB, the Hepcon Hemostasis Management System (HMS) Plus System (Medtronic International Ltd., Hong Kong) was used for automatic titration of heparin doses. CPB was conducted at a flow rate of 2.4 × body surface area L m^2 ^min^−1^.

### 2.2 Antimicrobial prophylaxis

**Figure 1:**
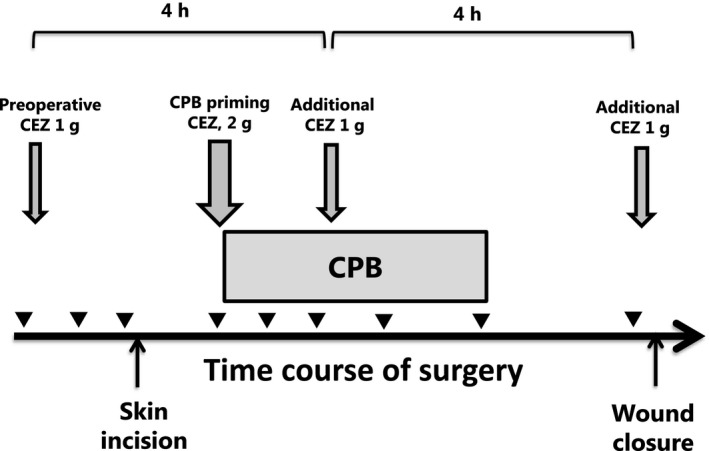
Cefazolin dosing and blood sampling schedules for patients undergoing cardiothoracic surgery with cardiopulmonary bypass (CPB). The down arrows (↓) represent intravenous administrations of cefazolin with doses (1 or 2 g), and the arrowheads (▼) represent blood samplings. The horizontal box represents the duration of CPB. The times of skin incision and would closure are indicated by up arrows (↑). In a patient whose course of surgery was complicated before CPB initiation, the second dose of cefazolin was administered before the initiation of CPB

Cefazolin sodium for injection (Nichiiko, Toyama, Japan) at a dose of 1 g was infused intravenously over 30 minutes. The infusion was started approximately 60 minutes before skin incision. Thereafter, additional dose was administered every 4 hours. In addition, 2 g of cefazolin was added to the priming solution of the CPB circuit. (Figure 1).

### 2.3 Blood Samplings

Blood samples were collected before the preoperative dose was given and 30 minutes after the completion of infusion. Blood samples were also taken immediately before and 30 minutes after the start and the end of CPB, and at wound closure. When an additional dose of cefazolin was needed for lengthy operation with CPB, blood samples were also obtained immediately before and 30 minutes after completion of the infusion of the supplemental dose. During CPB, blood samples (2.5 mL) for plasma cefazolin and albumin assays were withdrawn by anesthesiologists from an arterial catheter placed in the radial artery before and after CPB, and by medical engineers from the pump circuit. Blood samples were collected into serum separating tubes in which tetrahydrolipstatin (2.5 mg) had been added to a final concentration of 1 mg L^−1^. Tetrahydrolipstatin was added to blood samples for preventing in vitro lipolysis of triglyceride by heparin after sample collection.^15^, ^16^ Strictly speaking, serum was separated from blood samples taken before and during surgery until CPB was commenced, but plasma was separated from those taken during CPB and thereafter when patients’ blood was anticoagulated by heparin in vivo. For avoiding unnecessary complexity, we describe blood samples obtained by centrifugation of tubes as plasma hereafter. This is because our major interests were to study the disposition of cefazolin during and after CPB. Blood samples were centrifuged at 3000*g* for 10 minutes, and the separated plasma samples were kept in ice water bath until protein binding study. Plasma albumin concentrations were determined by the bromocresol green method using the A/G B‐test kit (Wako Pure Chemical Industries, Ltd., Osaka, Japan) according to the manufacturer's instructions.

### 2.4 Plasma cefazolin analysis

Total and unbound cefazolin concentrations in plasma were analyzed using a high performance liquid chromatography (HPLC) with UV detection method according to that reported by Nygard G. et al.^17^ with minor modifications. Briefly, for assay of total cefazolin, 100 μl of plasma and 200 μL of an internal standard solution containing cephapirin at 20 μg mL^−1^ in acetonitrile were mixed vigorously for 10 seconds, and then the mixture was centrifuged at 1700*g* for 5 minutes. Sixty microliters of the supernatant was mixed well with 340 μL of a 0.01 mol L^−1^ NaH_2_PO_4_ solution, and 50 μL of the mixture was injected into the HPLC column. The HPLC system employed in the present study consisted of a solvent delivery pump (Prominence LC‐20AD, Shimadzu Co., Kyoto, Japan), a TSKgel ODS‐80T_M_ column (5 μm, 4.6 mm i.d. × 150 mm; TOSOH, Japan) maintained at 40°C in a column oven (CTO‐20AC, Shimadzu), and an ultraviolet detector (SPD‐M20A, Shimadzu) set at 254 nm. The mobile phase consisted of a mixture of 0.01 mol L^−1^ NaH_2_PO_4_ and acetonitrile (88:12, in volume percent), and was used at a flow rate of 1.0 mL min^−1^.

For assay of unbound cefazolin, plasma samples were ultrafiltered with a Nanosep ultrafiltration device equipped with 10 K molecular weight cut‐off membrane (Pall Corporation, Port Washington, NY, USA). We performed plasma protein binding study within 12 hours after sampling. Plasma samples kept in ice water bath were warmed up at 37°C in a temperature‐controlled water bath for 10 minutes, and then were subject to ultrafiltration. Ultrafiltration was performed by centrifugation at 1000*g* for 20 minutes at room temperature. Fifty microliters of the ultrafiltrate and 50 μL of the internal standard solution containing cephapirin of 20 μg mL^−1^ in acetonitrile were mixed vigorously for 10 seconds, and then centrifuged at 1700*g* for 5 minutes. Sixty microliters of the supernatant was mixed well with 340 μL of a 0.01 mol L^−1^ NaH_2_PO_4_ solution, and 50 μL of the mixture was injected into the HPLC column. We found no significant absorption of cefazolin to ultrafiltration filters or device. The lower limit of detection with a signal to noise ratio of 3 or greater was 0.1 μg mL^−1^. Intra‐ and interday coefficients of variation of cefazolin assay were less than 5.8% and 6.5%, respectively.

### 2.5 Target plasma cefazolin concentrations

We set plasma unbound cefazolin concentrations of 2 and 8 μg mL^−1^ as the target thresholds for antimicrobial prophylaxis against SSI. These values are consistent with the breakpoints of Clinical and Laboratory Standard Institute (CLSI) for methicillin‐sensitive Staphylococcus aureus and susceptible Gram‐negative pathogens (e.g, Enterobacteriaceae).^1^, ^18^ Eight μg mL^−1^ of plasma unbound cefazolin concentration was used in a recent study as a target threshold level in patients undergoing cardiac surgery with CPB.^7^

### 2.6 Comparisons between measured plasma unbound cefazolin concentrations and those estimated using the Langmuir model

Decroix et al.^19^ revealed that cefazolin is bound exclusively to albumin and that binding is saturable. Using the Langmuir model (see below), they found that cefazolin has an affinity constant (*K*_a_) of 16 600 ± 1600 mol L^−1^ and one saturable binding site (n = 0.73 ± 0.02). The Langmuir model is formulated as follows:

(1) $$\frac{C_{b}}{C_{\mathrm{prot}}}=\frac{n\cdot K_{a}\cdot C_{u}}{\left(1+K_{a}\cdot C_{u}\right)}$$

where *C*_b_ and *C*_u_ are protein‐bound and unbound drug concentrations, respectively, at equilibrium; and *C*_prot_ is the concentration of binding protein. Since plasma protein binding of cefazolin has been shown to be attributed exclusively to albumin,^19^ *C*_prot_ may be replaced by plasma albumin concentration (*C*_alb_). Rearranging Equation 1 yields the following equation:

(2) $$C_{\mathrm{tot}}=\left(\frac{n\cdot C_{\mathrm{alb}}\times C_{u}}{K_{D}+C_{u}}\right)+C_{u}$$

where *C*_tot_ is total drug concentrations. Note that the dissociation constant (*K*_D_) equals 1/*K*a by definition. We assume that CPB alters plasma albumin concentration but not the binding affinity of albumin to cefazolin. We also assume that the equilibrium of drug‐protein binding is attained rapidly. Solving Equation 2 for Cu, the following equation is obtained^19^:

(3) $$C_{u}=\frac{1}{2}\times \left\{\left(C_{\mathrm{tot}}-n\times C_{\mathrm{alb}}-K_{D}\right)+\sqrt{{\left(C_{\mathrm{tot}}-n\times C_{\mathrm{alb}}-K_{D}\right)}^{2}+4\times K_{D}\times C_{\mathrm{tot}}}\right\}$$

By substituting *C*_tot_, *C*_alb_, and *K*_D_ into Equation 3, we estimated Cu for each plasma sample. Here, *C*_tot_ and *C*_alb_ were measured in the plasma samples collected from patients during surgery, and *K*_a_ was obtained from the literature.^19^ Then, we compared plasma unbound cefazolin concentrations (Cu) that were estimated by Equation 3 and those actually measured.

### 2.7 Statistical analyses

Demographic variables of the participants are presented as mean ± SD with range. Data of unbound fraction of cefazolin and plasma albumin concentration are presented using box and whisker plots. Nonparametric multiple comparisons of plasma unbound fraction of cefazolin and plasma albumin concentration before skin incision, during CPB, and at the end of CPB were performed using the Steel‐Dwass’ test for pair‐wise comparisons. Correlation between observed unbound cefazolin concentration and estimated concentration was analyzed by the least‐squares linear regression. A *P* value less than 0.001 was considered statistically significant.

## 3 RESULTS

### 3.1 Patient characteristics and adverse drug reactions

Twenty‐seven patients (18 males and nine females, aged 44‐93 years) were enrolled in the study and all subjects completed the study (Table 1). The mean CPB duration was 206 minutes (range: 129 to 305 minutes). None of them had major intraoperative complications or adverse drug reactions attributable to cefazolin and other drugs used during surgery. None of them developed SSI within 30 days after the operation.

**Table 1:** Demographic, laboratory and relevant cardiopulmonary bypass (CPB) parameters of patients

Characteristics	Data
Gender (male/female)	18/9
Age (years)	70 ± 12 [39‐93]
Body weight (kg)	62 ± 12 [42.8‐91]
Height (m)	1.60 ± 0.11 [1.44‐1.81]
Serum creatinine (mg dL^−1^)	0.89 ± 0.28 [0.48‐1.49]
Estimated GFR (mL min^−1^)	62.6 ± 19.6 [35.8‐113]
AST (IU L^−1^)	23 ± 7 [13‐42]
Perioperative patient data	
Surgical time (min)	428 ± 113 [253‐674]
Duration of CPB (min)	206 ± 51 [129‐305]
Priming volume for CPB (mL)	1310 ± 200 [1100‐1950]
Fresh frozen plasma (mL)	871 ± 979 [0‐4080]
Blood transfused (mL)	674 ± 725 [0‐2520]
Solution transfused (mL)	2450 ± 780 [1100‐4500]
Blood loss during operation (mL)	2180 ± 1290 [658‐5610]
Concomitant medications	Fentanyl, remifentanil, propofol

Data are shown as mean ± SD with range in brackets, or number of patients.

### 3.2 Plasma total and unbound cefazolin concentrations during surgery

**Figure 2:**
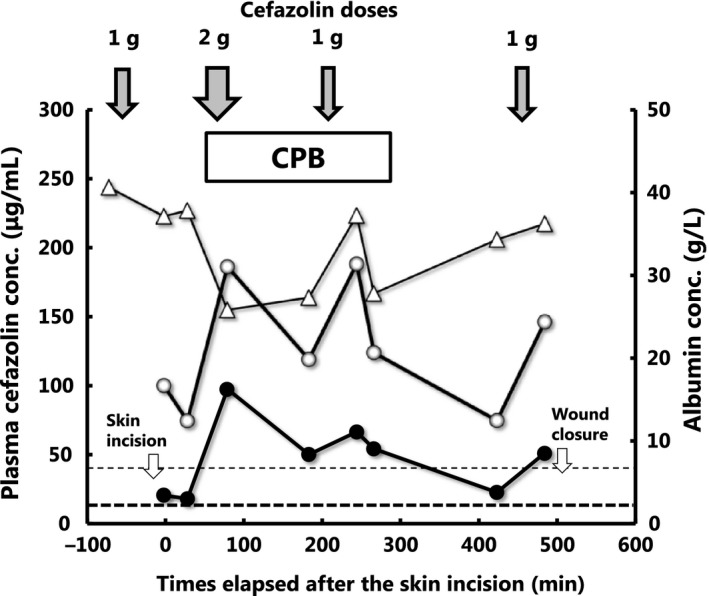
The time courses of plasma concentrations of total (○) and unbound (●) cefazolin (left vertical scale) as well as plasma albumin concentration (∆) (the right vertical scale) in a representative patient. The fine and bold horizontal broken lines represent target thresholds for total (40 μg mL^−1^) and unbound (8 μg mL^−1^) cefazolin concentrations for a representative *Staphylococcus aureus* strain. Down arrows at the top (↓) represent the doses and times of intravenous administration of cefazolin. The horizontal box represents the duration of cardiopulmonary bypass

**Figure 3:**
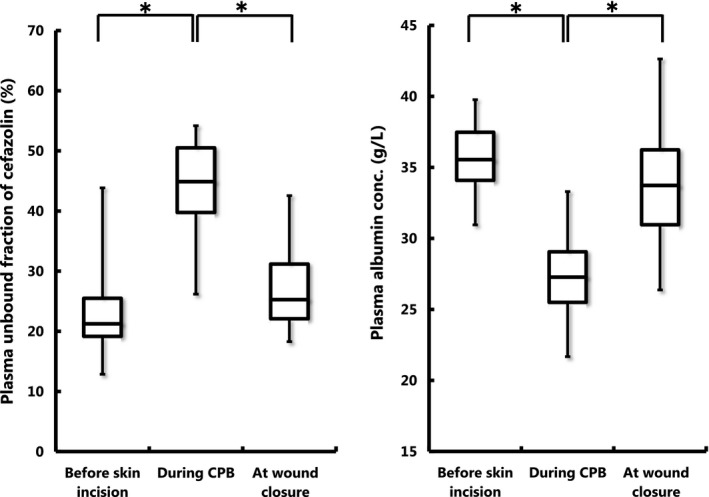
Box and whisker plots of plasma unbound fraction (%) of cefazolin (left panel) and plasma albumin concentrations (g L^−1^) (right panel) measured before initiation of CPB, during CPB, and at wound closure. The horizontal bands inside the box represent the medians, and top and bottom of the boxes are 75 and 25 percentile values, respectively. The ends of the vertical lines (whiskers) extending from the box upward and downward represent the maximum and minimum values, respectively. *P < 0.001 between the corresponding median values

The time courses of plasma (total and unbound) cefazolin concentrations and albumin concentration obtained from a representative patient before and during surgery are shown in Figure 2. The patient received four doses of cefazolin, since the operation took more than 8 hours. While all plasma unbound cefazolin concentrations measured throughout the surgery were higher than the target threshold for unbound drug (8 μg mL^−1^), trough unbound concentrations measured before skin incision, at the beginning of CPB, and before wound closure were close to the threshold concentration. Plasma total cefazolin concentrations were higher than 40 μg mL^−1^ throughout the surgical procedures. Plasma total and unbound cefazolin concentration obtained from patients immediately before skin incision, at the initiation of CPB or trough before CPB, during CPB, and at wound closure are shown in Table 2. Total and unbound cefazolin concentrations measured during surgery were almost all higher than the respective target concentrations. Especially, 99% (198 of 199 samples) of the plasma concentrations of unbound cefazolin measured during surgery were higher than 8 μg mL^−1^. Since plasma unbound fraction of the drug far exceeded 20% in samples obtained during and after CPB (Figure 3), a target threshold of total plasma concentration of cefazolin should have been considered lower than 40 μg mL^−1^. Nevertheless, 99% of total plasma cefazolin concentrations measured during surgery exceeded this value in our study.

**Table 2:** Plasma total and unbound cefazolin concentrations measured at different points during cardiothoracic surgery with cardiopulmonary bypass (CPB)

	Sampling time			
	Before skin incision (n = 27)	At initiation of CPB or trough before CPB (n = 29)	During CPB (n = 89)	At wound closure (n = 54)
Total concentration (μg mL^−1^)	79 [55‐134]	71 [29‐134]	159 [89‐232]	140 [60‐230]
% below target threshold (40 μg mL^−1^)	0	1^a^	0	0
Unbound concentration (μg mL^−1^)	17 [11‐35]	15 [6‐45.8]	70 [21‐137]	44 [13‐127]
% below 8 μg mL^−1^	0	1^b^	0	0
% below 2 μg mL^−1^	0	0	0	0

Data are shown as medians and ranges in the brackets.

^a^A patient with an irregular surgical course showed total and unbound plasma cefazolin concentrations of 35.4 and 8.3 μg mL^−1^, and ^b^another patient showed total and unbound plasma cefazolin concentrations of 40.4 and 6.0 μg mL^−1^, respectively. After receiving the first dose of cefazolin (1 g) before skin incision, initiation of CPB was delayed until 366 min from the first dose due to difficulties in operation, and he received the second dose of cefazolin before the initiation of CPB. The total and unbound plasma trough concentrations obtained immediately before the second dose (at 270 min from the first dose) were 40.4 and 6.0 μg mL^−1^, respectively. Total and unbound plasma drug concentrations measured at the initiation of CPB were higher than the respective target concentrations.

Plasma unbound fractions of cefazolin and albumin concentrations obtained before skin incision, during CPB, and at the end of CPB are shown in Figure 3. The median plasma unbound fraction of cefazolin measured before skin incision was 21%, but increased during CPB to 45%, and thereafter showed a tendency of returning to the basal level at wound closure (25%) (Figure 3). Significant (*P* < 0.001) differences were observed between before skin incision and during CPB, and between during CPB and at wound closure. In addition, the median plasma albumin concentration before skin incision was 36 g L^−1^, but decreased significantly (*P* < 0.001) during CPB to 27 g L^−1^, and increased again to 34 g L^−1^ at wound closure. Significant (*P* < 0.001) differences were observed between before skin incision and during CPB, and between during CPB and at wound closure (Figure 3).

### 3.3 Comparison between estimated and measured plasma unbound cefazolin concentrations

A total of 199 pairs of data set were available for this analysis. A significant (*P* < 0.001) linear correlation with a slope close to unity was observed between the estimated plasma unbound cefazolin concentrations (*C*_u,obs_) and the actually measured concentrations (*C*_u,measured_): *C*_u,obs_ = 1.05・*C*_u,measured_ + 1.49 (*r* = 0.86).

## 4 DISCUSSION

In the present study, we found that the median plasma unbound fraction of cefazolin showed an abrupt and profound increase during CPB (45%) compared to that at preoperative period (21%) (Figure**** 2). We speculate that these changes may be attributable to dilutional reduction of plasma albumin levels and saturable plasma protein binding of cefazolin.^20^ Especially, the CPB circuit was primed with approximately 1.3 L of crystalloid solution, and fresh frozen plasma was subsequently administered to compensate blood loss during operation (Table 1). Since cefazolin binds extensively and exclusively to albumin (80%‐86%),^6^, ^7^ the abrupt reduction in plasma albumin concentration during CPB may cause a corresponding increase in unbound fraction of the drug. In addition, as shown by Decroix et al.,^19^ the plasma protein binding of cefazolin is saturable at higher plasma concentrations. Marked increase of plasma cefazolin concentration was observed at the commencement of CPB due to the addition of 2 g of cefazolin to the priming solution of the CPB circuit. Our speculation may be supported by our finding of a good agreement between the unbound drug concentrations in plasma estimated using the Langmuir model and the concentrations actually measured. We estimated unbound cefazolin concentrations assuming that CPB affects the maximum binding capacity (i.e, plasma albumin concentration) but not the binding affinity of cefazolin binding. Nevertheless, we cannot completely exclude the possibility that heparin‐induced elevation of plasma free fatty acids is associated with the increased unbound fraction of cefazolin. Heparin is routinely used for anticoagulation during CPB, and it increases the activity of lipoprotein lipase in tissues and plasma, thereby increasing nonesterified (free) fatty acids concentrations. Fujiwara and Amisaki^21^ demonstrated that free fatty acids compete with drug binding at the warfarin‐binding site of human albumin. Cefazolin has been shown to bind to this site.^22^ While we added tetrahydrolipstatin during blood sampling to prevent in vitro lipolysis after blood sample collection, further studies are necessary to clarify the contribution of free fatty acids to altered plasma protein binding of cefazolin.

There has been an extensive debate over the target plasma unbound concentrations of cefazolin for the prevention of SSI. While 2 μg mL^−1^ may be enough against the most prevalent pathogen of methicillin‐sensitive Staphylococcus aureus (MSSA) and 4 μg mL^−1^ may be enough against a sensitive Enterobacteriaceae, 8 μg mL^−1^may be required for a resistant Enterobacteriaceae.^1^, ^7^ Holllis et al. adopted an inhibitory threshold of 16 μg mL^−1^ of unbound cefazolin for the validation of a dosing strategy for surgery requiring cardiopulmonary bypass.^23^ Recently, Zelenitsky et al reported that cefazolin plasma concentration during wound closure was associated with SSI at 30 days and that total cefazolin closure concentration of 104 μg mL^−1^ was a significant threshold for an increased risk of infection.^24^ In this context, we assessed the performance of our institutional regimen of cefazolin in reference to plasma unbound cefazolin concentrations of 2 and 8 μg mL^−1^, separately. While concurrent guidelines^1^ recommend administration of the first dose of cefazolin (1 or 2 g) within 60 minutes prior to skin incision and the second dose at wound closure, with or without supplemental doses (1 g) every 4 hours during operation, our protocol stipulates the administration of a supplemental dose (2 g) at the initiation of CPB (Figure 1). We demonstrated that our dosing protocol of cefazolin for antimicrobial prophylaxis used in our facility for patients undergoing cardiothoracic surgery with CPB (Figure 1) provides plasma unbound drug concentrations largely exceeding (99% of 199 samples) the target threshold of 8 μg mL^−1^ throughout the duration of surgery ranging up to 473 minutes (Figure 2 and Table 2). Recently, Calic et al^7^ evaluated the appropriateness of the traditional cefazolin regimen in patients undergoing cardiac surgery with CPB and found that plasma total cefazolin concentrations were lower than 40 μg mL^−1^ in 40% of intraoperative trough samples and 9.8% of samples obtained at wound closure. They estimated that plasma total cefazolin concentration of 40 μg mL^−1^ corresponded to an unbound drug concentration of 8 μg mL^−1^, assuming that plasma protein binding of the drug during surgery with CPB was the same as that of healthy subjects (80%‐86%).^6^, ^7^ Our study suggests that they might have underestimated the appropriateness of their protocol, since we observed a substantial decrease in plasma protein binding of cefazolin during CPB. Caffarellie et al^25^ also reported that the traditional dosing protocol of cefazolin was unable to maintain drug concentrations above 8 μg mL^−1^ in 50% of the patients when surgical time was longer than 120 minutes. De Cock et al^26^ performed population pharmacokinetic analysis on total and unbound plasma cefazolin concentrations in 54 children undergoing cardiac surgery with CPB. They concluded that an additional bolus dose at the start of CPB may improve the probability of target attainment from 59% to >94% in a typical pediatric patient. Collectively, the present study suggests that a supplemental dose of cefazolin at the initiation of CPB may be required for maintaining plasma unbound cefazolin concentration above the threshold level against typical Gram‐negative pathogens in adult patients undergoing cardiothoracic surgery with CPB. Obviously, further studies are required to seek for appropriate dosing regimens of cefazolin for pathogen‐specific target concentrations.

To establish an effective antimicrobial prophylaxis regimen with cefazolin for patients undergoing cardiothoracic surgery with CPB, we should develop a pharmacokinetic model that describes the disposition of plasma unbound drug during operation. The pharmacokinetics of plasma unbound cefazolin during CPB may be influenced by complex physiological changes associated with the procedure (including low serum albumin levels, transient increase in volume of distribution by connecting the CPB circuit to systemic circulation, changes in circulating volume by infusion and blood loss, and changes in liver and kidney perfusion).^5^, ^26^ The disposition of cefazolin may be particularly susceptible to alteration associated with CPB‐induced physiological changes, since the drug has a small volume of distribution (0.19 L kg^−1^ or 11.4 L per 60 kg) and binds extensively to albumin (80%‐86%).^6^, ^7^ Miller et al^13^ and Lehot et al^12^ studied the disposition of cefazolin in 8 and 10 adult patients undergoing surgery with CPB, respectively, and reported that the volume of distributions for total plasma cefazolin concentrations was increased during CPB, but they did not measure changes of unbound drug concentrations. Fellinger et al^27^ studied serum unbound cefazolin concentrations in a clinical setting similar to the present study, but they did not perform pharmacokinetic analysis. In this context, a pharmacokinetic analysis of unbound cefazolin concentrations in adult patients undergoing cardiothoracic surgery with CPB is definitely required. Such study is underway in our laboratory and the results will be reported elsewhere.

The present study has some limitations. Due to the small number of participants (n = 27) and lack of a comparator group receiving a conventional regimen, we cannot compare the probability of target attainment and the efficacy of antimicrobial prophylaxis between our protocol and the conventional regimen. In addition, we were unable to perform conventional pharmacokinetic analysis of total and unbound plasma cefazolin concentrations, since only a limited number of samples (n = 7 to 10) were available from each patient. We are examining the possibility of using our datasets to perform a population pharmacokinetic analysis, as mentioned above. For the prevention of SSI, it is important to determine the target site drug concentrations. Douglas et al measured the subcutaneous interstitial fluid (ISF) concentrations of cefazolin in patients undergoing elective/semielective abdominal aortic aneurysm open repair surgery and reported that the penetration of unbound drug from plasma to ISF was 85% (78% to 106%).^28^ Thus, the plasma concentration of unbound cefazolin would reflect the drug concentration at the target site.

In conclusion, our results indicate that altered plasma protein binding of the drug during and after CPB should be taken into consideration when modeling the disposition of unbound drug concentrations. Individualization of antimicrobial prophylaxis with cefazolin for adult patients undergoing CPB may be performed using a comprehensive pharmacokinetic modeling taking into account CPB‐induced physiological changes.

## DISCLOSURE

The authors have stated explicitly that there are no conflicts of interest in connection with this article.
